# The aggregation of Fe^3+^ and their d–d radiative transitions in ZnSe:Fe^3+^ nanobelts by CVD growth†

**DOI:** 10.1039/c7ra11356k

**Published:** 2018-01-15

**Authors:** B. B. Liang, L. P. Hou, S. Y. Zou, L. Zhang, Y. C. Guo, Y. T. Liu, M. U. Farooq, L. J. Shi, R. B. Liu, B. S. Zou

**Affiliations:** Beijing Key Laboratory of Nanophotonics and Ultrafine Optoelectronic Systems, School of Physics, Beijing Institute of Technology Beijing 100081 China 1297752298@qq.com houlipeng163@163.com zousy1987@126.com 18231193061@163.com 1019559512@qq.com 405542272@qq.com umairphysicist@yahoo.com ljshi@bit.edu.cn liuruibin8@gmail.com zoubs@bit.edu.cn +86-10-6891-8188

## Abstract

Transition metal (TM) doped II–VI semiconductors have attracted great attention due to their luminescence and diluted magnetism. In this study, the Fe^3+^-doped ZnSe nanobelts (NBs) were grown by a facile CVD method. The surface morphology observed *via* SEM is smooth and clean and the elemental composition measured *via* EDS confirms that the Fe^3+^ ions were incorporated into ZnSe NBs successfully. The micro-Raman scattering spectra demonstrate that the as-prepared NBs have the zinc blende structure. Furthermore, the Raman spectra of the Fe^3+^-doped NBs were compared with those of pure and Fe^2+^-doped reference samples. The former with a higher signal-to-noise ratio, an enhanced 2LO mode, a stronger LO mode redshift and a larger intensity ratio of LO/TO mode as well as the lower acoustic phonon modes confirms the better crystallization and the stronger electron–phonon coupling on Fe^3+^-incorporation. The emission of single Fe^3+^ ion, assigned to the ^4^T_1_ → ^6^A_1_ transition, was observed at about 570 nm. Moreover, increasing the doping concentration of Fe^3+^ ions caused the formation of different Fe–Fe coupled pairs in the lattice, which emitted light at about 530–555 nm for an antiferromagnetic-coupled pair, possibly due to the stacking faults and at about 620–670 nm for a ferromagnetic-coupled pair.

## Abstract 2

Transition metal (TM) doped II–VI semiconductors have attracted great attention due to their luminescence and diluted magnetism.

## Graphical abstract

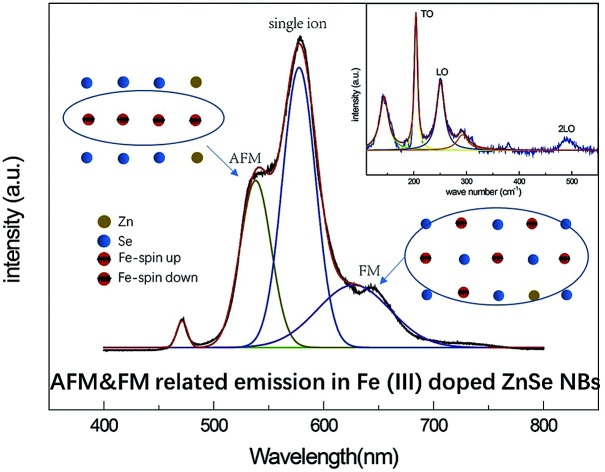

## Introduction

As a branch of diluted magnetic semiconductors, transition metal ion doped II–VI semiconductors have gained importance. The spin–spin coupling and the spin–carrier coupling of the host material and the active ion influences their semiconductor properties. In 2001, by theoretical calculation, Sato *et al.* found that electron doping in Fe(ii)-, Co(ii)- or Ni(ii)-doped ZnO could enhance the stabilization of the ferromagnetic state.^1^ Moreover, several researchers have used the co-dopant of magnetic ions and anions or cations with a valence different from the host material to introduce the free carriers for manipulating the electron spin,^2^ which is the basic concept for the design of spintronic devices. Simultaneously, the characteristic emission band of a transition metal ion was observed, which was derived from the splitting of the energy levels of free ions in the crystal field of the host material. Feng *et al.* have realized the lasing of Cr^2+^-doped ZnSe nanowires successfully for the first time.^3^ The tunable redshift of the Mn ion related to the d–d transition in the CdS lattice was also observed, in which the Mn ion aggregated *via* ferromagnetic coupling.^4,5^ It is also reported that the Mn ion antiferromagnetic pair emission near the stacking faults occurred in ZnSe:Mn nanoribbon with complicated electronic states and also, various properties were introduced.^6^ Furthermore, Bhattacharjee proposed the coupling of magnetic polaron associated with an electron–hole pair, which is called “EMP”.^7^ The EMP emission located at 460 nm was observed in ZnSe:Mn DMS nanoribbon^6^ and the EMP lasing has been observed in the Co(ii)-doped CdS nanobelts.^8^ Clearly, the interactions of the magnetic dopant and the host material strongly depend on the microstructures, the incorporation type, and the concentration. Therefore, they have impacts on the overall properties of the semiconductor.

ZnSe is a direct broad bandgap compound with 2.67 eV bandgap and a zinc blende structure under atmospheric pressure and room temperature.^9^ It has been extensively studied for its potential applications in blue-green light emitting devices and the first ZnSe based blue-green laser diodes were invented in 1992.^10^ The strong broad emission with lower energy than its bandgap is common, particularly in the low-dimensional nanostructures.^11–13^ The strong red emission at about 617 nm associated with the Zn-vacancy was observed in ZnSe nanowires.^13^ In addition, Sn-catalyzed tetrapod-branched ZnSe nanorod showed the as-mentioned emission contributed by the Zn vacancy, the interstitial states, the stacking faults, and the nonstoichiometric defects.^11^ Bukaluk *et al.* reported that the broadening of PL bands was due to the compositional and structural disorder.^12^ On the whole, different preparation conditions cause the formation of various local or extended defects and stacking faults in the ZnSe lattice, which hinders its wide application. Hence, the detailed formation processes, the structures, the composition characteristics, and the corresponding properties need to be studied.

Fe(iii), with a similar electronic configuration as Mn(ii), is seldom used for the DMS doping due to its larger p–d hybridization effect; also, its independent spin could not be easily maintained.^14^ Moreover, there is no explanation for the fact that Fe(iii) ion, unlike Mn(ii) ion, seldom functions as a dopant in semiconductors in terms of the luminescence *via* d–d transition. The recent findings on the iron compounds with superconductivity have indicated the clear carrier effect due to their strong p–d hybridization.^15^ In the present study, the Fe^3+^-doped ZnSe NBs are primarily investigated and compared with the pure and Fe^2+^-doped ZnSe NBs as the reference samples. The morphology of the Fe^3+^-doped NBs was observed by SEM and the element composition was analyzed by EDS. The micro-Raman and photoluminescence (PL) spectra of the as-discussed NBs were recorded to study their optical properties. Some novel properties have been identified in the Fe^3+^-doped ZnSe nanostructures. These findings will promote their future applications in the nanophotonic devices.

## Experimental

The Fe^3+^-doped ZnSe NBs were grown in a horizontal single-temperature zone furnace using the chemical vapor deposition (CVD) method, in which the mixture of ZnSe (Alfa Aesar, 99.99%, USA) and Fe_2_O_3_ (Aladdin, 99.9%, China) powders, used without further purification, served as the precursors and Au was used as the catalyst. A quartz tube was inserted into the furnace, following which the mixture with a molar ratio of 20 : 1 in a ceramic boat and the cleaned mica sheets sputtered with a 10 nm Au layer on another ceramic boat were loaded into the centre and downstream of the quartz tube, respectively. Subsequently, the high-purity gas mixture of 10% hydrogen and 90% argon was circulated through the tube at the rate of 50 sccm for 1 h to remove the air. Then, the temperature of the furnace was raised to about 1150 °C at the heating rate of 75 °C min^−1^ and kept at this value under the same conditions for 1 h. Eventually, the furnace was cooled down to room temperature naturally and the sample was dispersed on a cleaned silicon substrate. The pure and Fe^2+^-doped reference samples were prepared under the same conditions; FeCl_2_ was used as the precursor for the Fe^2+^-doped samples.

The morphology and elemental composition of the samples were characterized using a scanning electron microscope (SEM, Zeiss SUPRA 55, Carl Zeiss, Jena, Germany) equipped with an energy dispersive spectrometer (EDS, Zeiss SUPRA 55, Carl Zeiss, Jena, Germany), respectively. The optical properties of the samples were analyzed by recording the micro-Raman scattering and photoluminescence spectra, for which the 405 nm and 532 nm continuous-wave laser excitation sources were used, respectively. In addition to the light source, a confocal microscope (Olympus BX51M) and a spectrometer (Princeton SP2500) were used to converge and split the light into a spectrum; CCD (Princeton SP2500) was used as the light detector. Liquid nitrogen was used to reduce the temperature during the temperature-dependence spectroscopy tests. In addition, the magnetic response was measured *via* vibrating sample magnetism (VSM, LAKESHORE, 730T, America) technique.

## Results and discussion

**Fig. 1:**
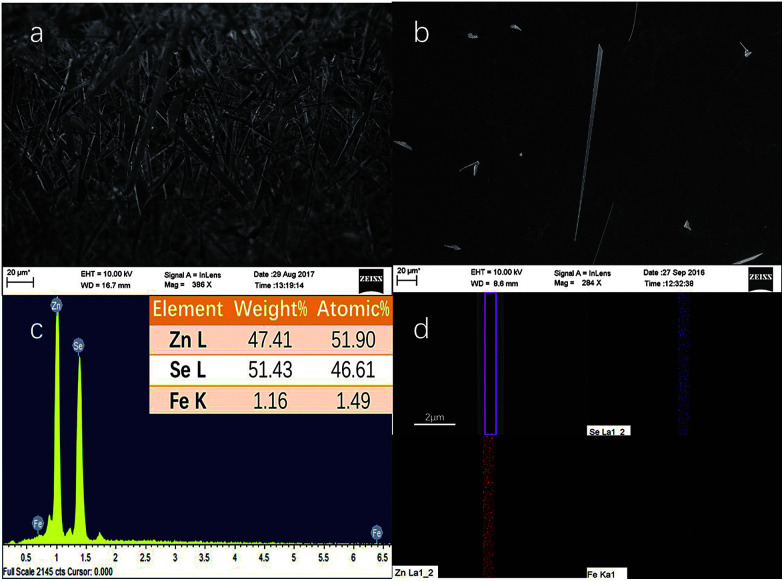
SEM images of (a) NBs grown on the mica sheet originally and (b) an individual Fe^3+^-doped ZnSe nanobelt dispersed on the cleaned silicon substrate. (c) Energy dispersive spectrum (EDS) of the as-dispersed nanobelt and the inset on the upper right is the elemental composition of the Fe^3+^-doped ZnSe NBs. (d) Energy dispersive spectra (EDS) mapping of Se, Zn, and Fe elements.

The SEM images of the samples originally grown on the mica sheet and an individual nanobelt dispersed on the silicon wafer are shown in Fig. 1(a) and (b), respectively. The morphology of the as-grown nanomaterial is nanowires, nanoribbons, or nanobelts with a smooth surface, which strongly depends on the growth temperature, the carrier gas rate, and the growth time. At the edges of the NBs, there are no metal balls visible to the naked eye, which is very common in this growth process. This proves that the formation mechanism of NBs is V-S and not VLS, which indicates that a slightly higher temperature than that for the gradual growth of nanowire is required. In addition, the width of most of the as-grown NBs reaches hundreds of nanometres or up to micron level with a 1D-like structure. The inset of Fig. 1(c) displays the elemental composition of NBs, which shows that the samples conform to the stoichiometric ratio and the doping of Fe element is achieved. Moreover, iron is the form of Fe(iii) instead of Fe(ii) because the valence state of the precursor is trivalent. Simultaneously, the mole ratio of the precursors has almost no influence on the morphology of the resultant nanostructure that is discussed in Chapter 2 of the ESI.†Fig. 1(d) is the energy dispersive spectra (EDS) mapping of Se, Zn, and Fe and the distribution Fe is far fewer than the other two.

**Fig. 2:**
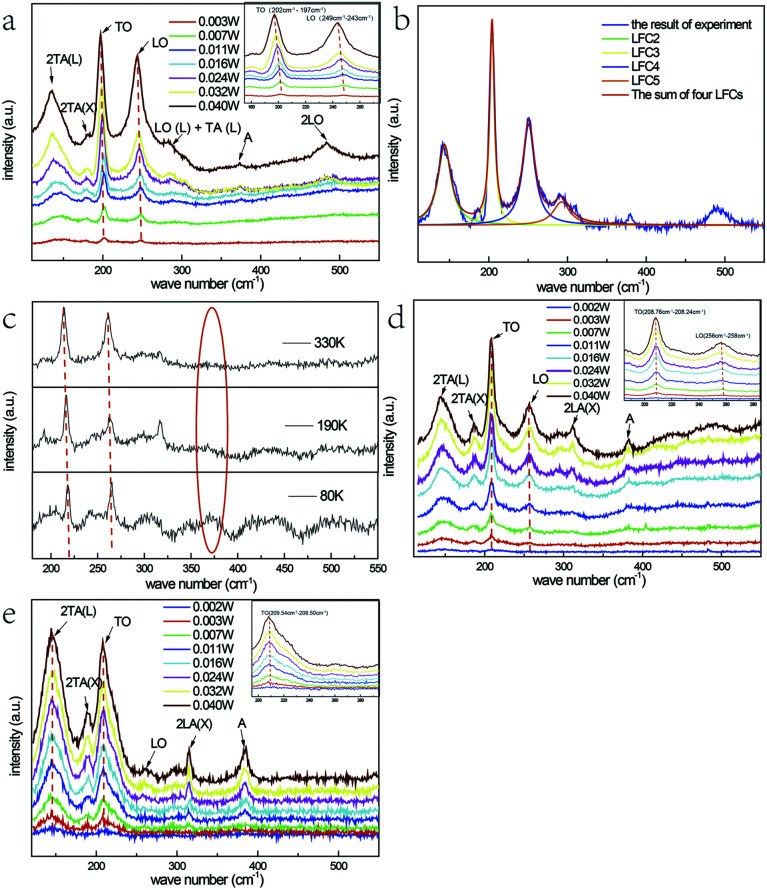
Room temperature micro-Raman spectra of an individual Fe^3+^-doped ZnSe nanobelt (a) with different excitation powers in the air, (b) at 0.032 W excitation power and Lorenz fitting curves for the labelled peaks, (c) at 70 K, 190 K, and 330 K in vacuum. Room temperature micro-Raman spectra with different excitation powers in the air of (d) an individual pure ZnSe nanobelt and (e) an individual Fe^2+^-doped ZnSe nanobelt.

Fig. 2(b) represents the room temperature micro-Raman spectra of the as-synthesized Fe^3+^-doped ZnSe NBs at 0.032 W excitation power in air; the spectra fit well with the Lorentz function curve. There are two known modes located at around 200 cm^−1^ and 245 cm^−1^ corresponding to the TO and LO phonons of ZnSe, respectively, which signifies that the as-grown NBs have the zinc blende structure. In addition, the peak locations of the above modes shift to a lower frequency in comparison with those of the bulk ZnSe crystal reported earlier because of the quantum size effect.^16^ The other four scattering peaks located at around 140 cm^−1^, 180 cm^−1^, 287 cm^−1^, and 485 cm^−1^ are labeled as 2TA(L), 2TA(X), LO(L) + TA(L), and 2LO, respectively, and they all belong to the higher-order phonon modes.^17^ This implies that there is strong anharmonicity in the lattice vibration. The formation of LO(L) + TA(L) occurs because the movement of some optical phonons is limited in the stacking faults related to the acoustic phonons. Fig. 2(a), (d) and (e) exhibit the micro-Raman spectra of the as-prepared Fe^3+^-doped ZnSe NBs, pure ZnSe NBs, and Fe^2+^-doped ZnSe NBs with an increase in the excitation power, respectively; all of the abovementioned measurements were performed in air and the measurement parameters were the same. It is clear that the Fe^3+^-doped ZnSe NBs possess a better signal-to-noise ratio than that of all other samples, which is a significant characteristic of good crystallinity for the zinc blende lattice. In addition, there are visible vibration modes located at around 310 cm^−1^ that are assigned to the structural defects,^17^ which is often modulated by the incorporation of dopants in the ZnSe lattice.

It is still disputable whether the weak scattering peak near 380 cm^−1^, labelled as A, is ascribed to the second order LO(X) + LA(X) mode^17^ or the oxidation state vibration.^18–20^ From Fig. 2(c), which exhibits the micro-Raman spectra of the as-prepared Fe^3+^-doped ZnSe single nanobelt detected at 80 K, 190 K, and 330 K in vacuum with 0.040 W excitation power, it is clear that there is no scattering peak observed at 380 cm^−1^ (noted by a red ellipse). This indicates that this scattering peak appears from the contact with air rather than the intrinsic quality of the Fe^3+^-doped ZnSe NBs. The similar vibration mode located near 380 cm^−1^ was once observed in ZnSeO_*x*_ alloy^19^ and ZnO,^20^ which confirms the incorporation of oxygen with laser heating. Simultaneously, the vacuum Raman spectra imply that the oxygen element of the precursor Fe_2_O_3_ has been exhausted in the growth and extracted out by the carrier gas. In addition, the Raman scattering peak 380 cm^−1^ only appears when the sample is excited with a relatively higher excitation power. As the power increases, the higher-order 2LO mode appears and becomes more distinct, while the same phenomenon cannot be observed in the vacuum Raman spectra. This indicates that the oxygen atom may involve in the formation of the 2LO mode, which has also been interpreted as D-centre caused by the O incorporation, which can cause the reduction of the bandgap^19^ and the enhancement of the multi-phonon process.^21^ However, when the Fe^3+^-doped ZnSe NBs are compared to the pure reference samples that were grown under the same condition (Fig. 2(d)), the 380 cm^−1^ peak intensity of the doped NBs is much lower than that of the pure NBs at the same excitation power, which indicates that the oxygen incorporation is harder in the doped ZnSe lattice than that in the pure samples. As the oxygen induced the Raman mode, the pure ZnSe NBs should exhibit the 2LO mode, similar to that in the doped NBs. However, the experimental results about the 2LO mode were not in accordance with the above expectation. Combining the above two comparisons, it can be concluded that the Fe^3+^ incorporation and the oxygen adsorption jointly promote the 2LO modes. However, the Fe^3+^ incorporation produces a stronger 2LO mode than that *via* O adsorption. Moreover, the existence of the Fe^3+^ ions suppress the O adsorption; thus, the 380 cm^−1^ mode in the Fe^3+^-doped nanobelt is much lower than that in the pure sample.

The frequency redshift and the intensity enhancement of the TO and LO phonon vibration modes of the Fe^3+^-doped NBs with the increase in the excitation power are shown in Fig. 2(a) (noted by red dotted lines). It is clear that the tendency of peak-shift with an increase in power input is in accordance with the tendency of the temperature-dependence variation (Fig. 2(c)). This indicates that the temperature enhancement caused by powers is one reason for the redshift. Moreover, it is notable that the variation of the locations of the LO- and TO-mode peaks in the Fe^3+^-doped sample is much larger than that in the pure and Fe^2+^-doped NBs under the same power. In addition, the LO- and TO-mode peak locations in the Fe^3+^-doped sample redshifted by about 4 cm^−1^, while those in the pure samples shifted by only 1 cm^−1^ and those in the Fe^2+^-doped NBs shifted by less than 1 cm^−1^. According to the study reported by Brajesh *et al.*, the peak position of the LO mode shows a downward shift, which is attributed to the electron–LO phonon coupling with an increase in the doping concentration.^22^ It means that the larger LO peak position red shift in Fe(iii) doped ZnSe NBs is related with the electron–LO phonon coupling. However, the TO-mode represents the intrinsic polar vibration of a local bond, whose peak-shift may arise from the stress effect or the vibration energy after the incorporation of oxygen or the Fe^3+^ ion. This is because the radius of the Fe^3+^ ion (0.67 Å), Zn^2+^ ion (0.74 Å), and Fe^2+^ ion (0.78 Å) is different; the radius of the Fe^3+^ ion is less than that of Zn^2+^ and one positive charge is left when Fe^3+^ replaces Zn^2+^ ion in the lattice. The Se–Zn bond may be relaxed when the Se–Fe bond is formed due to the structure or charge balance. This proves that the Fe incorporation has a much stronger influence on the lattice strength and order as well as the electron–phonon coupling.

Except for the above situation, it is noticeable that the intensity ratio of the LO/TO-modes of the Fe^3+^-doped ZnSe NBs is much larger than those of pure and Fe^2+^-doped NBs. The effect of Fröhlich electron–phonon coupling contributes to this phenomenon,^23^ in which the carrier movement in the lattice causes the ratio difference. It is known that the Fe^3+^ ion introduces positive charges that function as a possible carrier, while the Fe^2+^ ion introduces no charge inside the lattice. It can be observed that there are almost no LO- and 2LO-modes appearing in the spectrum shown in Fig. 2(e). However, the 2LA(X) and the oxidation state modes are prominent in the Fe^2+^-doped NBs. The latter phenomenon indicates that the Fe^2+^ ion at the Zn^2+^ site would facilitate the oxygen adsorption. Simultaneously, the carrier density does not increase and exhibit the electron–LO phonon coupling in its lattice. It is clear that the Fe^3+^-doped ZnSe NBs exhibits the highest electron–phonon coupling. Moreover, the electron–phonon coupling displayed by the pure reference sample is intermediate and that of the Fe^2+^-doped sample has the lowest. The effect of oxygen adsorption is in the opposite order. These characteristics influence their physical properties.

The difference in the intensities of the 2TA and 2LA acoustic phonon modes between the Fe^3+^-doped NBs and the reference samples is also distinct. The intensity of the Fe^3+^-doped sample is the lowest, the Fe^2+^-doped nanobelt is the highest, and the pure sample is intermediate. The acoustic phonons represent the collective shift relative to the mass-centre, which is hardly observed in bulk crystals. However, they could be enhanced in a small-size system. One example is the ZnSe nanowires or nanobelts grown by CVD. Due to the small difference in the energy value of ZnSe between wurtzite and zinc blende structures at high temperature, it is very easy to form the stacking fault and dislocations. This is the reason for the presence of the 2TA mode at around 140 cm^−1^ and the 2LA mode at 180 cm^−1^ in all types of ZnSe NBs. Such existence of the regular defect structures and anharmonic overtones in ZnSe nanowire or belts indicates the massive correlated defects. The different intensities of the above NBs is an interesting finding and the order is the same as that of their electron–phonon coupling magnitudes. Through cyclotron resonance, Langerak *et al.* proved that the electrons coupled to the LO phonon instead of the TO phonon,^24^ which supports our abovementioned arguments. In addition, the large energy mismatch between electron and acoustical phonons indicates the difficulty in coupling,^25^ but the collective contribution of an acoustic mode may strongly modify the longitudinal transport. Hence, the more stacking faults and the related higher acoustic mode can reduce the carrier mobility and hint the electron–phonon coupling further in the nanostructures. The enormous increase of the electron–LO phonon scattering rate had been observed in GaN in comparison with GaAs, which is ascribed to its much larger iconicity.^26^ The enhancement intensity of the LO-mode in these ZnSe nanostructures demonstrates the carrier propagation effect on the electron–phonon coupling.

**Fig. 3:**
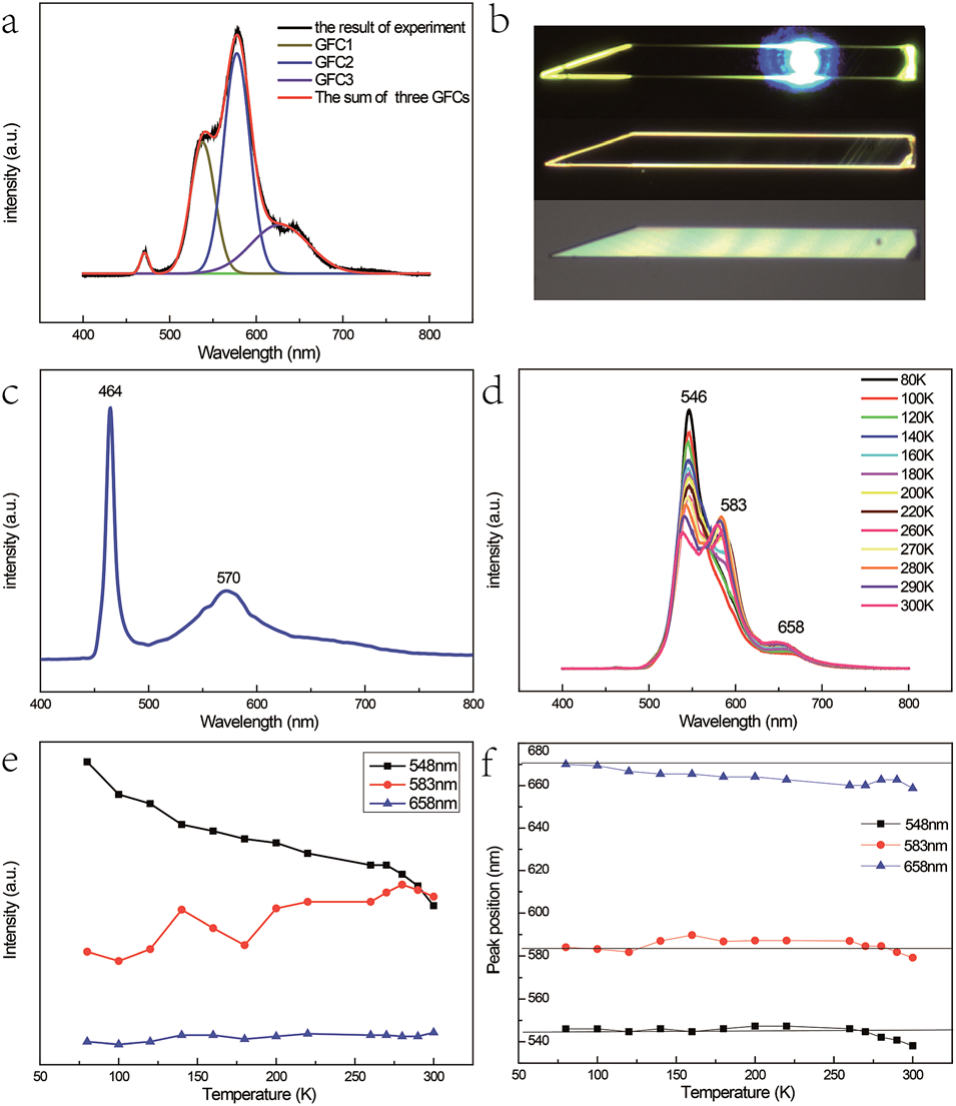
(a) The micro-photoluminescence (PL) spectra with Gaussian fitting curves of the as-prepared Fe^3+^-doped ZnSe NBs and (b) the corresponding optical images. (c) The PL spectrum prepared at a low temperature of 1100 °C and low Fe_2_O_3_ content with mole ratio of 40 : 1 and a short growth time of 0.5 h. (d) Temperature-dependence PL spectra of the as-prepared Fe^3+^-doped ZnSe NBs, (e) the corresponding variation in the emission intensity, and (f) the variation in the peak location.

Fig. 3(a) shows the micro-photoluminescence (PL) spectra of the as-prepared Fe^3+^-doped ZnSe NBs with Gaussian fitting curves and the corresponding optical images are shown in Fig. 3(b). Four emission peaks are detected; the peak with the highest energy is attributed to the near band edge emission. The origins of the other three peaks named GFC1, GFC2, and GFC3 are unclear since they have never been reported before. The FWHM of GFC1, GFC2, and GFC3 is 28 nm, 30 nm, and 66 nm and the peaks are located at around 538 nm, 577 nm, and 627 nm, respectively. This phenomenon is different from that observed in the pure and Fe^2+^-doped reference samples as discussed in Chapter 1 of the ESI.† In this case, the reference samples show a broad emission band at around 600 nm with a much larger FWHM. Some of these broad bands in the pure or doped ZnSe nanostructures are related to the point defects, the extended defects, and the stacking faults formed during their growth process.^11–13,27^ The deep level initiated by the Au catalyst in ZnSe may be another cause.^28^ However, in contrast to the deep level band with a variable energy primarily located at around 600–620 nm,^11,13,27^ the abovementioned three bands have different energy ranges. In addition, the oxygen adsorption state, which is related to the laser illumination conditions, cannot lead to the abovementioned emission bands either at its energy level lying at the band edge or at higher energy levels.^29^ Considering the entire situation, we think that these emission bands are derived from the d–d transitions of the Fe^3+^ ions in the ZnSe lattice. The emission band range also verifies that the doping ions are not divalent because the d–d transition of Fe^2+^ in ZnSe related emission is in the infrared range.^30^

As discussed in the above section, the micro-Raman spectra of the as-prepared Fe^3+^-doped ZnSe NBs with the zinc blende structure indicate that the Zn^2+^ site has the tetrahedral (Td) symmetry and the replacement of the Zn^2+^ ion by the Fe^3+^ ion stabilizes the ZnSe lattice. When the Fe^3+^ ion with a 3d^5^ configuration was incorporated into the Zn^2+^ site of ZnSe NBs, the ground state ^6^S and the first excited state ^4^G of free Fe^3+^ ion split into ^6^A_1_, and ^4^T_1_, ^4^T_2_, double degenerate ^4^A_1_, and ^4^E levels, respectively, under the impact of the Td crystal field; the same splitting appeared in other host materials.^31–33^ As a result, one conjecture that the three emission bands arising from the d–d transitions, namely, ^4^T_1_ → ^6^A_1_, ^4^T_2_ → ^6^A_1_, and ^4^E → ^6^A_1_ are notable. It is known that the 3d^5^ configuration of the Fe^3+^ ion is the same as that of Mn^2+^. The optical absorption spectra in Cd_0.6_Mn_0.4_S single crystals displayed a similar emission profile at 455 nm, 480 nm, and 510 nm due to the above mentioned d–d transitions of Mn^2+^ (ref. 34). However, the energy difference between ^4^T_1_ and ^4^T_2_ of Mn^2+^ does not match well with our results. The possibility of such transitions is also small because two higher levels only radiate under the condition of ferromagnetic coupling in the lattice.^8,35^ Hou *et al.* prepared the Fe^3+^-doped ZnSe nanobelts successfully with two emission bands at 553 nm and 630 nm, respectively, which are attributed to the phenomenon of the transitions of ^4^T_2_ → ^6^A_1_ and ^4^T_1_ → ^6^A_1_.^36^ The different emission bands displayed by our as-prepared sample were similar to that reported by Begum *et al.*, which is due to the site symmetry of the Fe^3+^ ion in the host ZnSe material, which is identified as octahedral.^37^ Hence, the abovementioned assignment is not plausible.

**Fig. 4:**
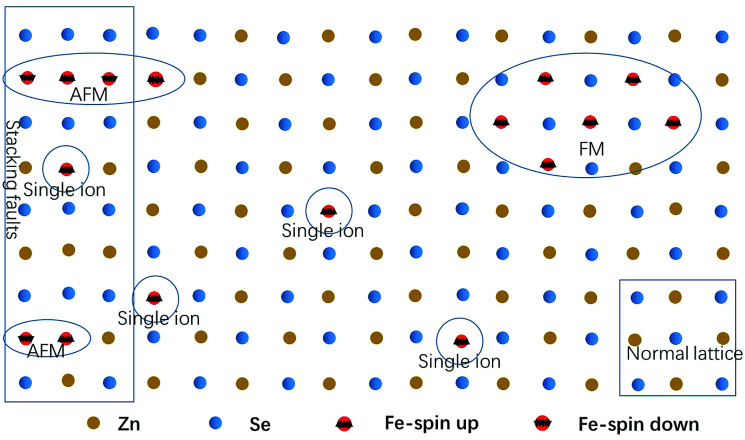
The simplified diagram of AFM and FM.

**Fig. 5:**
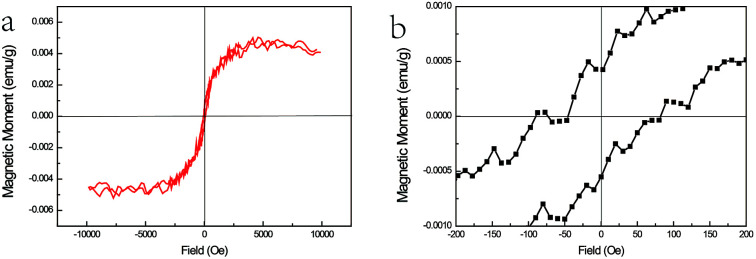
(a) The *M*–*H* curves of ZnSe:Fe^3+^ belts at 300 K and (b) the magnified area near the zero magnetic field.

As the content of the precursor (Fe_2_O_3_), the growth temperature, and the growth time are reduced, the PL spectra (Fig. 3(c)) can be obtained with the distinct emission band usually located at 565–585 nm. In fact, the higher growth temperature, the longer growth time, and the higher Fe precursor content contribute to the higher dopant concentration to a certain degree, which has been discussed in Chapter 2 of the ESI.† The emission band is related to Fe^3+^ and it agrees with the results of the Fe^3+^-doped CdS NBs, in which the orange light emission has been observed and the bands were located at around 573 nm.^38^ We ascribe the photoluminescence to the ^4^T_1_ → ^6^A_1_ transition. There is no doubt that the concentration of Fe^3+^ becomes lower under this preparation condition and the variation of the dopant concentration will affect the photoluminescence properties of the NBs as shown in Fig. 3(a) and (c). The competition between the near band edge and the Fe^3+^-related luminescence is apparent, in which the increasing Fe^3+^ concentration lowers the intensity of the near band edge and the same situation has been observed in ZnSe:Mn QDs.^39^ In addition, comparing to that observed for the low Fe^3+^ concentration, the two peaks shown in Fig. 3(a) at the higher energy and lower energy side of ^4^T_1_ → ^6^A_1_ transition emission are remarkable. The d–d transition of TM ion related emission appears in numerous systems^30,40^ with a premise that there are no same neighboring TM ions in the range of one wavelength around the dopant ion to avoid the resonant energy transfer between TM ions. Recently, we found that the ferromagnetic or antiferromagnetic (MnX)_*n*_ cluster can exhibit an emission of d–d transition nature.^5,6^ The same electron configuration of Mn^2+^ and Fe^3+^ makes it possible to infer that these two peaks arise from the antiferromagnetic coupling pairs (AFM) and ferromagnetic coupling pair (FM) of the Fe^3+^ ions, which is in agreement with that reported for ZnSe:Mn nanoribbons.^6^ The simplified diagram of the formation of AFM and FM is shown in Fig. 4. Fe(iii) related compounds can easily form antiferromagnetic states.^41,42^ In addition, the ferromagnetic Fe–Fe coupling in the normal lattice is already verified by magnetic response measurement (Fig. 5). The TM ion-cluster in a typical host semiconductor crystal would be stabilized in the ferromagnetic state, while its own bulk crystal displays the antiferromagnetic state. The presence of two peaks indicates that their origin is related to the amount of Fe^3+^ ions in the lattice. The increase in the Fe^3+^ ion concentration easily causes the aggregation and the magnetic ion pair leads to the ferromagnetic coupling at high temperature, which is common in numerous DMSs.^8^ And the ferromagnetic coupling pairs related emission ranges from 620 nm to 670 nm in as-prepared NBs. Simultaneously, the antiferromagnetic state appears in the vicinity of the stacking fault layer, of which the existence is conceived by the above mentioned Raman spectra. The microscopic optical techniques are used to study the origin of magnetism in the Fe^3+^-doped ZeSe nanobelts, which is an important way to find a novel function of DMS.

Fig. 3(d) shows the PL spectra of the Fe^3+^-doped ZnSe NBs at various temperatures (different sample from Fig. 3(a)). One visible feature is that the AFM related emission peak drops faster than the other two peaks with the increase in temperature as shown in Fig. 3(e). This phenomenon conforms to the formulation that the Fe–Se–Fe AFM pairs are in the vicinity of the stacking faults related to the acoustic phonon vibrational mode. The high temperature causes the higher electron–acoustic phonon coupling, which leads to the abovementioned situation in the emission spectra. It can also explain the almost fixed FM state related emission due to the fact that the coupled Fe ions are located on the normal lattice site and not on the defect sites. In addition, the single ion d–d transition emission becomes subtly higher with the rising temperature, which is possible because some high energy AFM states have relaxed to this state. The ^4^T_1_ → ^6^A_1_ transition is forbidden by the symmetry and spin selection rules, which is not in accordance with the result. This is because of the sp–d hybridization effect^43,44^ of the dopant and host materials. Moreover, the variation in the location of the FM related emission peak confirms the inference. Eventually, the variable emission band in the observed range is also caused by the p–d hybridization combining with definite covalence. In fact, the d–d transition emission of the Fe^3+^ ion in ZnSe strongly depends on not only the ion lattice location, the neighbor ion, the symmetry, and the aggregation, but also the carrier effect and the lattice relaxation due to the electron–phonon coupling. This CVD preparation method under the current experimental conditions can only realize trace doping since the concentration of Fe(iii) cannot often exceed the critical value due to the segregation. Furthermore, the excessive doping is discussed in Chapter 3 of the ESI.†

The magnetic response of ZnSe:Fe^3+^ nanobelts was measured at room temperature with the magnetic field ranging from +1 to −1 T as shown in Fig. 5(a). The *M*–*H* curves represent the magnetic hysteresis loops, which are related to the ferromagnetic behaviour. In addition, the ferromagnetic response is derived from the high spin state of Fe(iii) in the ZnSe lattice with the largest magnetic moment (*g* = 5/2) among the TM ions. The magnified area near the zero magnetic field indicates that the coercive field is about 100 Oe. The overall magnetism is not large, which may be related to the influence of the antiferromagnetic pairs. Moreover, this magnetic measurement matches with the PL spectra results, confirming that the ferromagnetic-coupled pair exists and contributes to this magnetism.

## Conclusions

Overall, the Fe^3+^-doped ZnSe NBs were grown by a simple CVD method and different test methods demonstrated that Fe^3+^ was doped into ZnSe NBs. The iron ion-doping in ZnSe introduces, the surplus free carriers and the micro-Raman scattering spectra show the different features of the as-prepared NBs in comparison with the reference samples. The better signal-to-noise ratio, the lower acoustic phonon modes (2TA and 2LA), and the oxygen-related vibration modes at 380 cm^−1^ along with the appearance of the 2LO modes confirm that Fe^3+^ promotes the good crystallinity for the zinc blende lattice. Moreover, the LO mode exhibits the largest frequency red-shift and the highest intensity ratio of the LO/TO modes, thus indicating the strong electron–phonon coupling. The PL spectra show a clear Fe^3+^-related internal d–d transition emission, which is assigned to the ^4^T_1_ → ^6^A_1_ transition of a single Fe^3+^ ion. In addition, the emission related to the antiferromagnetic and ferromagnetic coupling occurs at the higher-energy and lower-energy sides of single Fe^3+^ ion, respectively, with an increase in the ion concentration. The temperature-dependence PL spectra indicate that the p–d hybridization and electron–phonon coupling have a significant impact on the Fe^3+^ ion related emission. This is the first report on the d–d transition emission of the Fe^3+^ ion doped on the Zn^2+^ site in ZnSe. However, numerous properties of the Fe^3+^-doped ZnSe NBs, as one of the TM doped II–VI semiconductors, need to be explored. In addition, the ZnSe:Fe(iii) QD is promising to have a strong emission, similar to that of ZnSe:Mn QDs.

## Conflicts of interest

There are no conflicts to declare.

## Supplementary Material

RA-008-C7RA11356K-s001
