# Supplementary material for: The aggregation of Fe3+ and their d–d radiative transitions in ZnSe:Fe3+ nanobelts by CVD growth
Source: RSC Adv. 2018 Jan 15;8(6):3133–9. doi: 10.1039/c7ra11356k (PMC9077547; doi:10.1039/c7ra11356k)
Supplement: RA-008-C7RA11356K-s001 [file RA-008-C7RA11356K-s001.pdf]

Electronic Supplementary Material (ESI) for RSC Advances.
This journal is © The Royal Society of Chemistry 2017

Supporting information

1. The PL spectura of pure and Fe^{2+}-doped ZnSe NBs

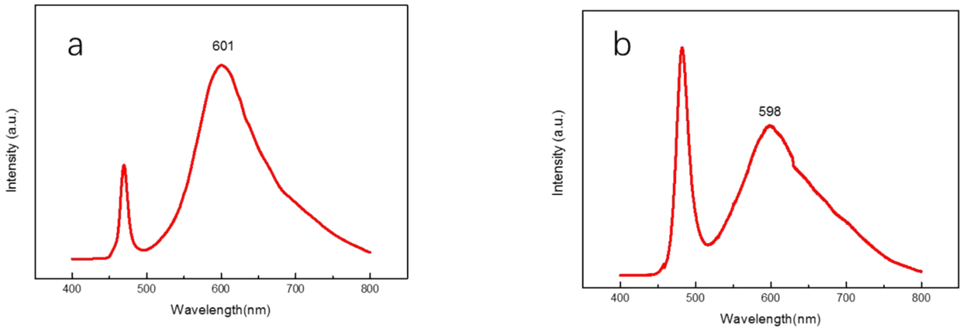

Figure 1. (a) The micro-photoluminescence (PL) spectra of pure ZnSe NBs at 3.2 mW excitation power; (b) Fe^{2+}-doped ZnSe NBs micro-photoluminescence (PL) spectra at 3.2 mW excitation power.

2. The influences of growth conditions on NB properties

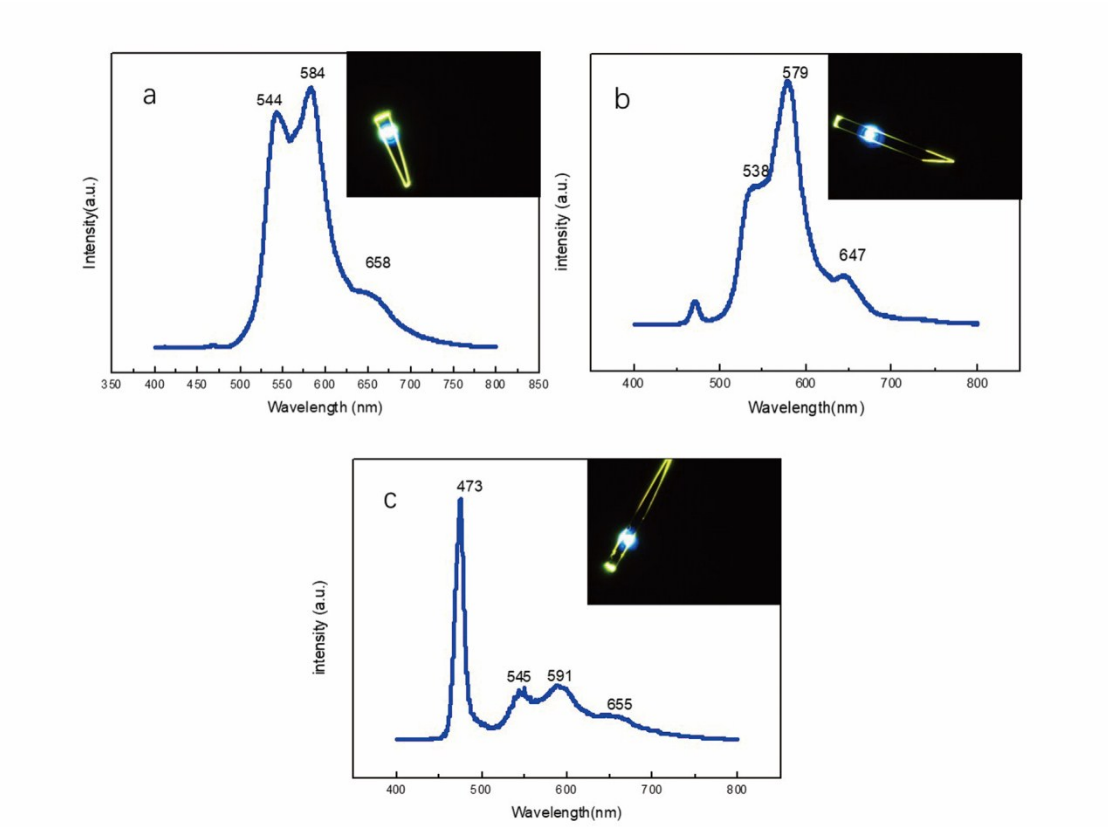

Figure 2. The PL spectra prepared under 1150℃ for 1h with mole ratio of (a)10:1, (b)20:1, (c)40:1. The gas rate is 50sccm with heating rate of 75°C/min. The insets on the top right are the optical microscopy images of NBs with the same magnification.

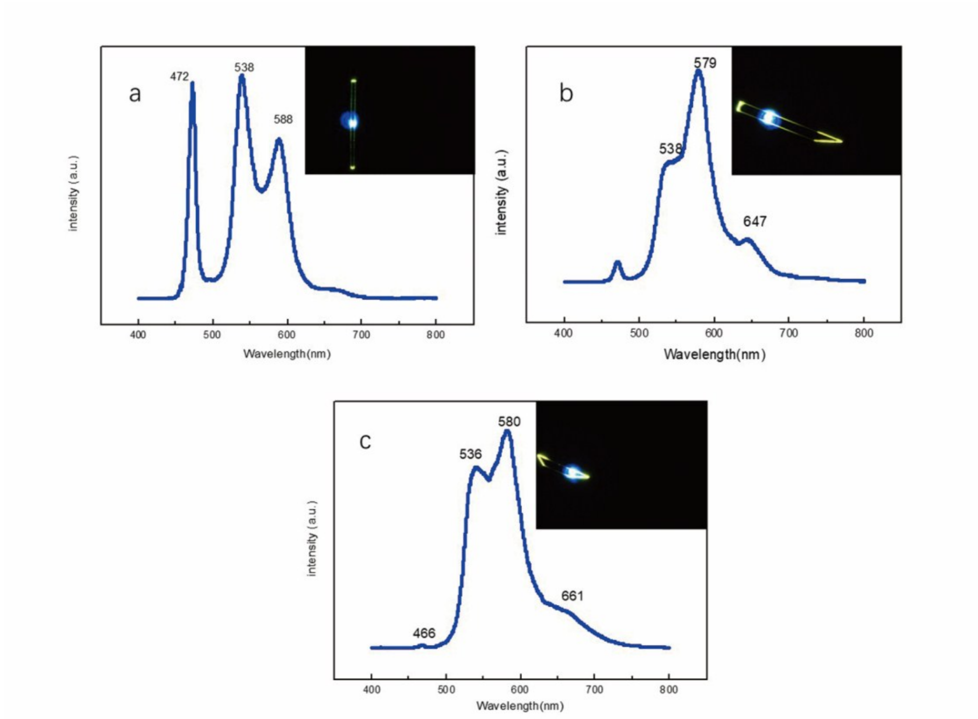

Figure 3. The PL spectra of NBs prepared under (a)1150℃, (b)1100℃ for 1h with mole ratio of 20:1, the gas rate of 50sccm, heating rate of 75℃/min; (c) The optical microscopy images of NBs with the same magnification at the top left, the top right, the bottom corresponding to preparation temperature 1150℃, 1100℃, 1050℃.

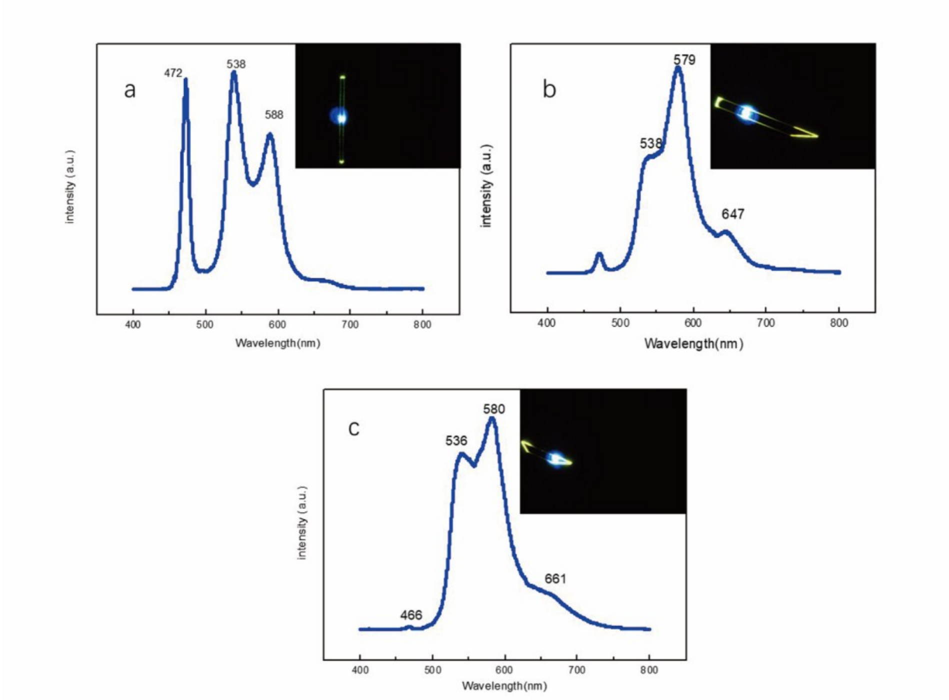

Figure 4. The PL spectra of NBs prepared under 1150℃ for (a) 0.5h, (b) 1h, (c) 1.5h with mole ratio of 20:1, the gas rate of 50sccm, heating rate of 75℃/min; The insets on the top right are the optical microscopy images of NBs with the same magnification.

Figure 2,3 and 4 shows the influences of precursors ratio, growth temperature and growth time on the morphology and photoluminescence of NBs. It is clear from the optical microscopy images that the growth temperature has great impact on the sample size and morphology. And the optical signal of NBs prepared at 1050℃ is too weak to be detected. The growth time has influence on the morphology but precursors ratio almost has no influence on the morphology. We focus on their optical responses but not the morphology in this manuscript. The competition process between the near band edge and the dopant-related luminescence has been found in ZnSe:Mn QDs in which the increasing Mn(II) concentration made the intensity of near band edge relatively reduced[1] and the same situation happens in our product. And combine with Figure 2,3 and 4, it can be deduced that the higher growth temperature, the longer growth time and the more Fe precursors ratio contributes to the higher dopant concentration. Figure 3(b) shows the single ion related d-d transition emission and its intensity will become weaker if the growth time and precursors ratio decrease. In a word, the formation of antiferromagnetic and ferromagnetic coupled pair is related with Fe^{3+} aggregation, and the incorporated ion concentration is one of influencing factor of Fe^{3+} aggregation that can be changed by growth temperature, growth time and precursors ratio, but in a way not simplified model due to different ion aggregation and segregation. Take into account the temperature range of the furnace of us and ZnSe evaporation and deposition, the growth temperature range 1100℃~1150℃ is suitable that is relatively narrow. The higher temperature in above range usually causes more defects while the more trace doping of Fe^{3+} offsets part of the defects according to the results of Raman spectra. So the quality of resultant product is affected by temperature in narrow range while it is affected by comprehensive factors. Longer time or higher temperature may cause black areas in the belt, which contain more carrier effect and show weak emission even no emission. The ion aggregation and carrier effect has related to the FM and AFM coupled pair, both may contribute to the ferromagnetism[2, 3]. And we presented optical study on the coexistence of AMF and FM pair in this Fe(III) doped DMS.

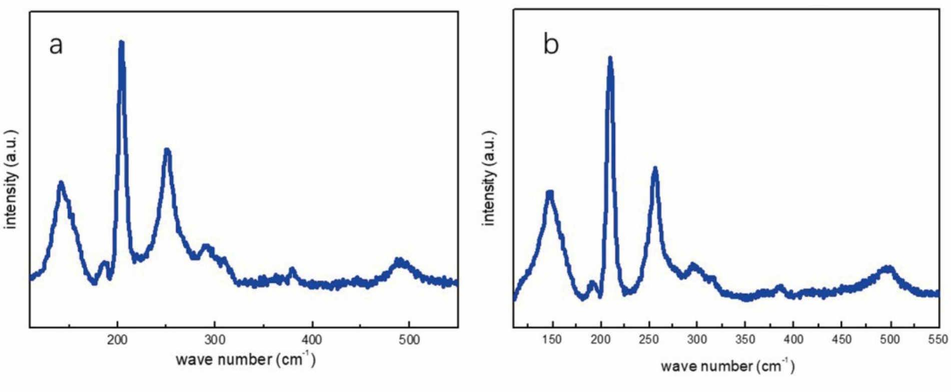

3. The excessive doping

Figure 5. The micro-Raman spectra of an individual Fe^{3+}-doped ZnSe nanobelt

prepared under (a) 1150℃ for 1h with mole ratio of 20:1; (b) and 1100℃ for 0.5h with mole ratio of 40:1. The gas rate is 50sccm with heating rate of 75℃/min.

Fe^{3+} doping level is related with precursor ratio, growth temperature and growth time. And under our current laboratory conditions, we can only do the trace doping while if the concentration of dopant exceeds the critical value by other methods, according to Dietl et al[4], we think excessive ions may lead to the formation of new Fe compound phase. And under our current laboratory conditions, the variation of Fe^{3+} content usually is too little（<1.5%）,there is no much difference about electron-phonon coupling and lattice strength shown in Figure 5. For the PL measurement, sometimes there is inhomogeneous optical phase in the belt if the concentration of dopant exceeds the critical value, it is observed that excessive ions lead to heavy carrier effect or even the formation of new Fe compound, which give weak and broad emission shown in Figure 6(a) or even can't emit light (black color) shown in Figure 6(b), but not from d-d transition. While some other area of belt in Figure 6(b) can emit light (light color). For the magnetic response, the higher concentration the larger of magnetic susceptibility, but this is not the subject of this manuscript.

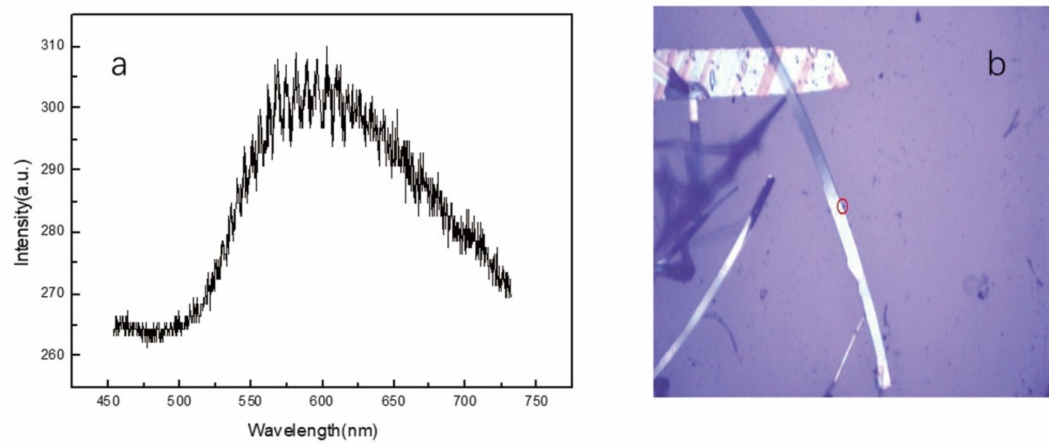

Figure 6. The emission spectra of high dopant concentration area of doped belts and the optical morphology of samples. The black area or belts give minor or no emission when excited.

references

1. S. Mahamuni, A. D. L. And and S. Patole, *Journal of Physical Chemistry C*, 2008, **112**, 2271-2277.
2. A. Walsh, S. J. Da and S. H. Wei, *Phys. Rev. Lett.*, 2009, **102**, 256401.
3. Z. Sun, W. Yan, G. Zhang, H. Oyanagi, Z. Wu, Q. Liu, W. Wu, T. Shi, Z. Pan and P. Xu, *Phys.rev.b*, 2008, **77**, 5208.
4. T. Dietl, T. Andrearczyk, A. Lipińska, M. Kiecana, M. Tay and Y. Wu, *Phys.rev.b*, 2007, **76**.
